# Case Report: Comprehensive management of trichilemmal carcinoma of the lower leg in an elderly patient with comorbidities: a case study on integrated ALA-PDT and surgical excision

**DOI:** 10.3389/fmed.2025.1546359

**Published:** 2025-03-14

**Authors:** Chan Hu, Xiaojing Liu, Mingshun Wu, Caihe Liao, Yan Zhao, Mingyuan Xu, Yongxian Lai, Guolong Zhang

**Affiliations:** ^1^Institute of Photomedicine, Shanghai Skin Disease Hospital, Tongji University School of Medicine, Shanghai, China; ^2^Department of Dermatopathology, Shanghai Skin Disease Hospital, Tongji University School of Medicine, Shanghai, China; ^3^Department of Dermatologic Surgery, Shanghai Skin Disease Hospital, Tongji University School of Medicine, Shanghai, China

**Keywords:** dermatology, trichilemmal carcinoma, ALA-PDT, immunomodulatory, surgical resection

## Abstract

Trichilemmal carcinoma (TC), a rare malignancy originating from hair follicle cells, typically affects sun-exposed skin in older individuals and necessitates differential diagnosis from other skin carcinomas. Here, we present one case of trichilemmal carcinoma in a 92-year-old female, admitted to our hospital with a large exophytic red tumor on her right lower leg. Notably, the patient had a history of long-standing hypothyroidism and hypertension, indicating that she was unable to tolerate extensive excision. Histopathology showed outer root sheath hyperplasia with clear cell differentiation and keratinization, alongside increased atypical cells. Immunohistochemistry revealed CK7 and EMA positivity, confirming follicular origin, while negative CK10, CK18, CEA, CK20, and GCDFP-15 excluded other follicular tumors. A high Ki-67 (80%) indicated high proliferative activity, supporting the TC diagnosis. Treatment involved managing her underlying conditions, followed by ALA-PDT and surgical excision for the carcinoma. This case illustrates the challenges in managing trichilemmal carcinoma in elderly patients with multiple comorbidities, emphasizing the importance of a comprehensive approach to treatment.

## 1 Introduction

Trichilemmal carcinoma (TC) is an uncommon malignant adnexal neoplasm originating from hair follicle cells (1). Typically, lesions appear predominantly on the face and occasionally on other sun-exposed areas among the elderly population. However, in rare cases, TC may arise in non-sun-exposed regions and exhibit an aggressive growth pattern. Clinically, TC presents as an asymptomatic exophytic or polypoid mass, occasionally featuring scales, ulceration, or rolled borders. Therefore, it is necessary to distinguish it from squamous cell carcinoma, basal cell carcinoma, keratoacanthoma, or proliferating pilar cyst. While TC may exhibit local invasiveness, its progression often follows an indolent trajectory if managed with complete excision. Surgical excision is typically the primary treatment for TC, with limited reported alternative therapies beyond surgery (2). Wide surgical excision provides thorough local treatment but results in large wounds, slow recovery, and often requires skin grafting, making it suitable for patients with good surgical tolerance. Radiation therapy, while effective for patients who are not candidates for surgery, may cause chronic radiation dermatitis and other complications, and is typically used for cases of postoperative recurrence. In comparison, local excision combined with ALA-PDT offers a less invasive approach, leading to quicker wound healing and fewer side effects. Despite the need for multiple treatments and its limitation to superficial lesions, ALA-PDT stands out as an ideal option for elderly patients or those with poor surgical tolerance, offering a safer and more efficient treatment alternative. Additionally, due to the prevalence of underlying health conditions and complexities in elderly patients, combined treatments are sometimes necessary. Therefore, we report a case of a 92-year-old female diagnosed with a large TC on her right lower leg, treated with combined ALA-PDT and surgical excision. In this case, the tumor was significantly larger than typical TC and exhibited exophytic growth, posing substantial clinical challenges. Additionally, the patient’s advanced age and multiple comorbidities further increased the complexity of treatment.

## 2 Case report

**Figure 1:**
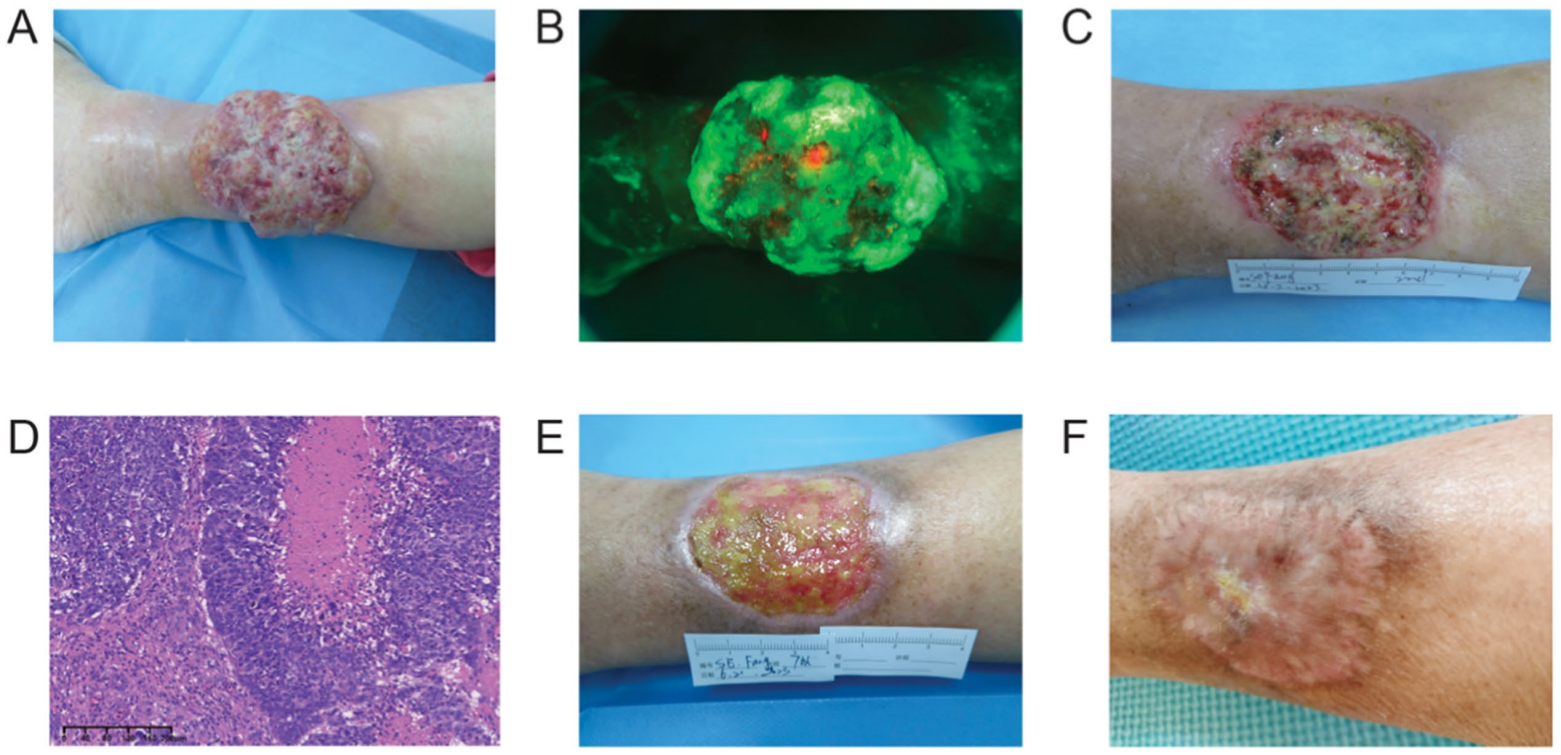
**(A)** Upon admission. **(B)** ALA fluorescence in the wound. **(C)** After surgical excision. **(D)** The histology results. **(E)** After 6 post-surgical sessions of ALA-PDT. **(F)** After 1 year follow-up, the wound healed well.

A 92-year-old woman presented with a large exophytic red tumor on the anterior surface of her right lower leg for 6 years, which had rapidly enlarged and ulcerated over the past 2 years, causing significant pain. The patient had a history of hypertension for over 30 years, previously reaching blood pressure levels of 160/100 mmHg, controlled with bisoprolol. Additionally, she had been on levothyroxine (50 μg/day) for hypothyroidism for more than 50 years. A dermatological examination revealed a 10 × 8 cm oval-shaped red exophytic tumor with irregular edges, surface erosion, and exudation on the right lower leg (Figures 1A,B). The mass was firm and bled easily upon palpation. No enlarged superficial lymph nodes were detected. Ultrasound examination of the tumor on the right lower leg showed a central thickness of approximately 28 mm and a subcutaneous thickness of about 5 mm. The tumor had a hypoechoic interior, an irregular shape, and clear margins at the base, with adjacent swollen subcutaneous soft tissue. Color Doppler Flow Imaging (CDFI) revealed rich blood flow signals inside the tumor and a 5 mm wide subcutaneous vein beneath it. There was no enlargement of the bilateral inguinal lymph nodes. Pathological examination of the tumor showed local ulceration and necrosis. In the dermis, there were multiple clusters of basophilic cells, numerous dyskeratotic cells, significant cellular atypia, increased mitotic figures, transparent-like cells in certain regions, and collagen proliferation and sclerosis in the stroma. Immunohistochemistry showed CK7+, CK20−, GCDFP-15−, CK10−/CK18−, CK19+, EMA+, CEA−, SMA−, with a Ki-67 (80%+), consistent with a diagnosis of proliferative trichilemmal carcinoma (Figure 1D).

After thorough communication with the patient, due to the large size of the lesion, advanced age, and multiple underlying conditions, the patient refused the surgical excision (wide excision) option and accepted photodynamic therapy. Therefore, for the large, malodorous tumor with significant secretion, a single session of neoadjuvant ALA-PDT was administered to control infection and stimulate the local immune response. The treatment involved the application of 20% 5-ALA cream (Shanghai Fudan-Zhangjiang Biopharmaceutical Co., Ltd.™, Shanghai, China) on the tumor surface, and incubated under dark conditions for 0.5 h. Subsequently, the tumor was irradiated with the light-emitting diode (LED) red light (630 ± nm) (Kernel Medical Equipment Co., Ltd.) at a power density of 40 mW/cm^2^ and an energy density of 150 J/cm^2^.

Due to the exophytic growth pattern of the lesion, surgical excision of the majority of the visible tumor lesions, including a 2 mm margin, was performed under local infiltration anesthesia with 1% lidocaine (Figure 1C). Post-surgery, when the wound showed no significant exudation, additional ALA-PDT was carried out to eliminate subclinical lesions that are difficult to identify with the naked eye. This involved ALA-PDT treatments on the subclinical lesions for a total of six sessions treatments with a two-week interval between each session (Figure 1E). Each PDT involving the application of 20% ALA cream incubated for 0.5 h and irradiation (200 J/cm^2^, 84 min). The lesion progressively improved with each PDT session, and the wound gradually healed completely. Throughout the treatment period, the patient experienced minimal discomfort and functional impairment. ALA-PDT was well tolerated, with only mild burning sensations and transient erythema, without severe pain or systemic side effects. The limited surgical excision avoided the need for skin grafting and extensive postoperative wounds, facilitating smoother recovery. The patient was followed up remotely via WeChat due to advanced age, limited mobility, and residing in another province, as she was unwilling to travel for in-person hospital visits. Follow-up evaluations were conducted through patient-provided digital photographs and virtual consultations every 3 months. During the 12-month follow-up, the patient remained recurrence-free, with good wound healing and no significant mobility restrictions (Figure 1F). This outcome further supports the feasibility of ALA-PDT as a viable alternative in elderly patients with large TC lesions who are not suitable candidates for extensive surgery. The comprehensive treatment timeline was summarized in Table 1.

**Table 1:** Treatment timeline.

Time point	Treatment phase	Interventions	Objectives
TP0 (baseline)	Diagnostic phase	Skin biopsy, Immunohistochemistry, and ultrasound evaluation	Confirm TC diagnosis and assess the depth of invasion
TP1 (pre-op day 5)	Neoadjuvant therapy	1 session of ALA-PDT (20% ALA, 150 J/cm^2^, red light 630 nm)	Control infection and reduce tumor size
TP2 (day of surgery)	Surgical excision	Excision with 2 mm margins around the lesion	Complete removal of visible tumor
TP3-TP8 (post-op 2–12 weeks)	Adjuvant therapy	6 sessions of ALA-PDT (200 J/cm^2^, once every 2 weeks)	Eradicate residual lesions, reduce recurrence risk, and promote wound healing
TP9 (post-op 12 months)	Follow-up	Follow-up of communication and photo documentation via WeChat	Monitor recurrence and wound complications

## 3 Discussion

Trichilemmal carcinoma (TC) is a rare malignant tumor originating from the outer root sheath of hair follicles, primarily characterized by differentiation towards the outer root sheath. Clinical manifestations typically include solitary keratinized nodules resembling polyps, cauliflower-like growths, or ulcerations, often misdiagnosed as squamous cell carcinoma, basal cell carcinoma, or keratoacanthoma (3). TC predominantly affects elderly individuals, with a higher incidence in sun-exposed areas such as the scalp and face. However, the present case is unusual due to several factors: (1) The lesion was exceptionally large compared to most reported cases, posing a significant clinical challenge. (2) The patient was an elderly female (92 years old) with multiple comorbidities, complicating treatment decisions. (3) The tumor developed on the lower leg, a relatively rare site for TC, as approximately 90% of cases are reported in sun-exposed regions. These factors highlight the atypical presentation of TC in this case, emphasizing the need for individualized treatment approaches, particularly in elderly patients with extensive lesions and limited surgical options.

Although TC is generally low in malignant potential, its histological features and biological behavior may vary, with potential for invasiveness but slow growth and minimal recurrence or metastasis. Treatment typically involves surgical excision, with a recommended safety margin of 1 cm (2). Apart from surgical excision, there is a lack of reported alternative treatment options. Given the patient’s advanced age, multiple comorbidities, and the tumor’s size and exophytic growth, extensive surgical excision was deemed high-risk. Thus, ALA-PDT was chosen as a safer, less invasive alternative.

Histologically, TC is characterized by proliferating epithelial cells forming follicular sebaceous gland-like structures, appearing lobulated (4). The tumor cells consist of squamous epithelial cells and clear cells, with disorganized arrangement, marked pleomorphism, and visible mitotic figures. Central abrupt keratinization within the lobules forms keratin cysts, with reddish-stained amorphous material in the cystic cavities. Peripheral epithelial cells form a palisading arrangement around the tumor mass, with fibrous stromal tissue proliferation observed around the tumor periphery. Cytologically, characteristic features include dysplastic clear cell components and trichilemmal keratinization, which connects with the epidermis and follicular epithelium. The most important feature is the sudden keratinization without granular cell layers, distinguishing it from keratin pearls in squamous cell carcinoma. Squamous cell carcinoma, also originating from epithelial cells, lacks lobulated epithelial cell proliferation and exhibits high Ki-67 expression correlated with malignancy degree.

Photodynamic therapy (PDT) is a non-scarring cancer treatment method that involves applying 5-aminolevulinic acid (ALA), which is converted into a photosensitizer, protoporphyrin IX (PpIX), and subsequently activated by visible light. Beyond its direct cytotoxicity, ALA-PDT influences the tumor microenvironment by modulating immune responses. One study has reported that it can reduce IL-10 and IL-6 levels, which may help limit tumor growth and spread (5). Also, it was shown that it could affect Th2 cytokines like IL-4, IL-13, IL-31, and IL-33, strengthening immune surveillance and lowering the risk of recurrence (6). This suggests ALA-PDT not only kills tumor cells but also helps sustain long-term tumor control. However, its limited penetration depth (2–3 mm) can restrict its effectiveness for large tumors (7). Therefore, surgical excision of most of the visible tumor lesions was performed to reduce the lesion thickness, thereby enhancing ALA penetration and improving the treatment effect. New adjuvant therapies, such as preoperative ALA-PDT, are used to reduce lesion size, control infection, and activate the immune response, thereby creating a suitable environment for subsequent treatments. While PDT has been widely used for nonmelanoma skin cancers, its role in TC management remains underexplored. Beyond its cytotoxic effects, PDT has been suggested to exert immunomodulatory functions, potentially enhancing the immune response against residual tumor cells. In this case, the neoadjuvant application of ALA-PDT helped control infection and reduced tumor burden before excision, while adjuvant PDT was utilized to target subclinical lesions, ensuring a more comprehensive treatment approach. In elderly patients, especially those with large lesions who cannot tolerate extensive surgery, and those with multiple comorbidities, this combined approach offers a viable alternative. For these patients, extensive surgical excision is often not feasible due to the potential for poor wound healing and high recurrence rates. While effective, its limitation lies in its penetration depth of only 2–3 mm, which may not be sufficient for treating TC lesions thicker than 5 mm. Therefore, surgical debulking of the tumor to the minimum depth before PDT is beneficial for more thorough treatment, ensuring better efficacy for deeper lesions.

In our case, the patient, being of advanced age with significant comorbidities, and the tumor’s enormous size along with exophytic growth and limited invasion into the basal layer, presented a unique challenge. Standard extensive excision would be difficult because it typically requires a longer surgical time and results in a larger skin defect. Therefore, skin grafting is usually necessary postoperatively. This procedure is particularly challenging for tumors located on the lower leg. The combined approach of limited excision followed by PDT offered a practical solution. Post-surgical debulking of the tumor, PDT was employed to treat the entire cancerous area, ensuring thorough removal of potential malignant cells and promoting wound healing.

The integration of surgical intervention with ALA-PDT highlights the importance of tailored and continuous care, taking into account the patient’s overall health and ability to withstand treatment. Combining surgical excision with ALA-PDT therapy offers a comprehensive approach to effectively manage giant TC, serving as a viable alternative treatment that achieves outcomes comparable to extensive resection. This strategy not only guarantees complete tumor removal but also utilizes PDT to address any remaining or recurring lesions, while promoting wound healing, thereby presenting a holistic and tailored treatment solution. Regular follow-up using dermoscopy and skin ultrasound is essential to monitor for recurrence, although advanced age may limit the patient’s ability to attend frequent follow-ups. Importantly, a 12-month follow-up showed no signs of recurrence, further supporting the potential efficacy of this strategy in TC management. The long-term control observed in this case highlights the feasibility of ALA-PDT as an alternative approach, particularly for elderly patients with large lesions who are not ideal candidates for extensive surgical resection.

## 4 Conclusion

This case highlights the successful management of a giant trichilemmal carcinoma in an elderly patient with multiple comorbidities using a combined approach of limited surgical excision and ALA-PDT. Given the tumor’s large size and unusual location on the lower leg, as well as the patient’s poor surgical tolerance, this strategy minimized surgical trauma while effectively reducing tumor burden and preventing recurrence. The successful 12-month recurrence-free follow-up suggests that ALA-PDT, when combined with limited excision, can effectively manage large TC in elderly patients with surgical limitations. Further studies are needed to validate its long-term efficacy and optimize treatment protocols.

## Data Availability

The original contributions presented in the study are included in the article/supplementary material, further inquiries can be directed to the corresponding authors.
